# Impact of 1,6-hexanediol on *Schizosaccharomyces pombe* genome stability

**DOI:** 10.1093/g3journal/jkad123

**Published:** 2023-06-07

**Authors:** Chance E Jones, Susan L Forsburg

**Affiliations:** Section of Molecular & Computational Biology, University of Southern California, Los Angeles, CA 90089, USA; Section of Molecular & Computational Biology, University of Southern California, Los Angeles, CA 90089, USA

**Keywords:** genome stability, 1,6-hexanediol, phase separation, *Schizosaccharomyces pombe*, heterochromatin, nucleolus, rDNA, H3K9me3

## Abstract

Phase separation is a major mechanism of macromolecular condensation within cells. A frequently chosen tool for global disruption of phase separation via weak hydrophobic interactions is treatment with 1,6-hexanediol. This study evaluates the cytotoxic and genotoxic effects of treating live fission yeast with 1,6-hexanediol. We find that 1,6-hexanediol causes a drastic decrease in cell survival and growth rate. We also see a reduction in HP1 protein foci and increase in DNA damage foci. However, there is no evidence for increased genomic instability in two classically phase-separated domains, the heterochromatic pericentromere and the nucleolar rDNA repeats. This study reveals that 1,6-hexanediol is a blunt tool for phase separation inhibition and its secondary effects must be taken into consideration during its in vivo use.

## Introduction

Phase separation is the process of protein condensation due to weak and reversible domain/motif binding of various proteins (Brangwynne et al. 2015; Alberti 2017; Shin and Brangwynne 2017). These weak and reversible bonds are often transient and can be brought about by long stretches of intrinsically disordered or unstructured regions that can have a variety of binding partners (Fasting *et al*. 2012). These condensates can also be brought about by weak hydrophobic protein domain interactions such as from phenylalanine-glycine repeats (Ribbeck and Görlich 2002; Patel *et al*. 2007). These processes are often aided by another physical process known as gelation which can further concentrate and compartmentalize proteins (Harmon *et al*. 2017). Instead of relying upon lipid bilayer membranes that require energy and transport mechanisms, or upon simple diffusion that requires too many proteins to be produced to be economical, cells utilize the physical chemistry of the proteins themself to self-concentrate where needed without any energy expenditure.

**Table 1.** Strain list.

Fy261	h + leu1-32 ade6-M216 ura4-D18 can1-1	Forsburg and Nurse 1994
FY528	h + his3-D1 ade6-M210 ura4-D18 leu1-32	Liang *et al*. 1999
FY1520	h90 ura4-DS/E leu1 YIP2.4 pUCura4-7	Thon and Verhein-Hansen 2000
FY4101	h + nmt1(41X)-GFP-nhp2::leu1* his7-366 ade6-M210 ura4-D18 leu1-32	Maiorano *et al*. 1999
FY4267	h − nmt1(41X)-GFP-nhp2::leu1* sad1-mCherry::Ura4 + gar2-mCherry-KanR leu1-32 ura4-D18 his5- ade6-M210	This study
FY5187	h − ade6-Δ ura4-D18 leu1-32 his1-102 ChL[ubcp4::LEU2::chk1 hph:spccB3.18 spcc1322.09::ura4 + ade6+]	Li *et al*. 2013
FY5546	h + gar2-mCherry::kanR rad11-Cerulean::hphMX rad22-YFP::natMX leu1-32 ura4-D18	This study
FY8900	h + Swi6-GFP::kanMX6 Chp1-mCherry::natMX6, leu1-32 ura4-D18 ade6-M210	This study
FY9279	h + nmt1(41X)-GFP-nhp2::leu1* Δswi6::kanMX ade6-M210 ura4-D18 leu1-32	This study

There are many examples of phase-separated, nonmembrane-bound nuclear organelles including paraspeckles, Cajal bodies, PML bodies, stress granules, and the nucleolus (Handwerger *et al*. 2003; Mao *et al*. 2011; Banani *et al*. 2017; Shin and Brangwynne 2017). These regions allow concentration of various functions and activities in sub-nuclear domains. The nucleolus is the largest of the phase-separated nuclear organelles and is the center of rRNA biogenesis (Feric *et al*. 2016). In higher eukaryotes there are multiple nucleoli per cell found scattered about the nucleus (Pederson 2011). In eukaryotes with smaller genomes such as yeast, there is only one large nucleolus that is located opposite the centromere/spindle pole body (SPB) (Matsuda *et al*. 2017). Unlike other phase-separated intracellular organelles, the nucleolus is further phase-separated internal sub-regions. The innermost region, the fibrillar center (FC), is responsible for initial rRNA transcription and contains the highest concentration of RNA PolI. The dense fibrillar complex (DFC) is responsible for initial rRNA processing such as trimming and modifications. The granular component (GC) is generally responsible for the final maturation of rRNAs and is the outermost region in contact with the general nucleus (Feric *et al*. 2016). This tripartite phase separation of the nucleolus in these higher eukaryotes also relies upon the nucleophosmin/nucleoplasmin (NPM) group proteins, which facilitate the multivalency needed for nucleolar phase separation (Mitrea *et al*. 2018). In simple eukaryotes such as yeast, there are only two general regions of the nucleolus, the FC and a combined region, consisting of the functions of the DFC and GC (Thiry and Lafontaine 2005).

Heterochromatin domains are also phase separated (Strom *et al*. 2017; Tatarakis *et al*. 2017). Heterochromatin is an epigenetically delimited chromatin state that is transcriptionally repressed. Classically, these regions are marked by tri-methylation of histone H3 lysine 9 (H3K9me3), which creates a binding site for chromodomain-containing proteins including the HP1 proteins, conserved in many eukaryotes (Bannister *et al*. 2001; Lachner *et al*. 2001). In the fission yeast *Schizosaccharomyces pombe* (*S. pombe*), Swi6 is an HP1 family member that has been shown to phase separate in vitro and in vivo in the presence of H3K9-methylated chromatin (Sanulli *et al*. 2019). Aggregation of Swi6-bound regions into droplet is presumed to concentrate Swi6 isolate heterochromatic domains from the rest of the chromatin. This may contribute to three-dimensional nuclear localization and topologically associated chromatin domains.

Phase separation via weak hydrophobic interactions can be disrupted by treatment with general compounds such as the aliphatic alcohol 1,6-hexanediol (Ribbeck and Görlich 2002; Patel *et al*. 2007; Kroschwald *et al*. 2015; Peskett *et al*. 2018; Ulianov *et al*. 2021). While useful in vitro, many studies do not consider potential for broader cytotoxic effects in vivo. In this study, we examined the in vivo response of fission yeast cells treated with 1,6-hexanediol and found it is toxic to cell survival and detrimental to cell growth even at very low concentrations. We observed partial fragmentation of the nucleolus, with significant disruption of the localization of Swi6 specifically in heterochromatin domains but not other H3K9me3 binding proteins. We found that treatment with 1,6-hexanediol increases the number of Rad52 and RPA foci in the nucleus, leading us to conclude that there is an increase in general genome instability upon treatment, but this was not localized to nucleolar or heterochromatin domains. We conclude that 1,6-hexanediol increases general genome instability and cytotoxicity. Thus, it is not indicated for targeted disruption of locally phase-separated regions.

## Material and methods

### Cell growth and physiology

Fission yeast cell growth and physiology were matched to previous lab protocol described in Forsburg and Rhind (2006) and Sabatinos *et al*. (2012). Strains used can be found in Table 1.

### Cell survival and growth rate

In order to measure cell survival on 1,6-hexanediol, *S. pombe* cells were incubated at 32°C in 10 mL of rich yeast extract supplemented (YES) media for 24 h to mid log phase. The cells were then treated with the corresponding concentration of 1,6-hexanediol. Samples for the 24-h time point were diluted ½ with more YES media to compensate for increased growth over the longer time point. Cells were then counted using a hemocytometer, and 500 cells were plated onto YES media using glass beads for spreading. Colonies were then counted and ratioed against untreated cells.

To calculate growth rate in 1,6-hexanediol, *S. pombe* was incubated in 5 mL of rich YES media overnight. Each replicate was counted using a hemocytometer, and 5 × 10^6 from each were divided into 100 mL of rich YES media in 250-mL flasks with either no 1,6-hexanediol or the corresponding concentrations described in figure. Cells were then incubated for 12 h. OD at 595 nM was then checked at T = 12 to T = 24. OD ratios were calculated to the WT OD of that corresponding replicate.

### Live cell imaging

Methods for live cell imaging are adapted from Forsburg and Rhind (2006) and Green *et al*. (2015). *S. pombe* cells were taken from plates and grown overnight in 5 mL of rich YES media. They were then spun down and washed once with 1 × PBS, and a portion of cells were incubated in PMG-HULALA (PMG + Histidine, Uracil, Leucine, Adenine, Lysine, and Arginine, 225 mg/L each) at 32°C overnight (Sabatinos and Forsburg 2010). Upon reaching mid log phase, cells were treated with either no 1,6-hexanediol or the corresponding percentage outlined in each figure. Cells were then spun down after the described about of time and placed on 1% agarose/PMG-HULALA pads that were made at least 1 h prior allowing them to dry slightly. Pads were then covered with a coverslip and sealed with VaLap (1/1/1 w/w/w Vaseline/lanolin/paraffin) and imaged directly. During long-term timelapse imaging, a small gap of roughly 1–2 mm in length was left unsealed using VaLap. Fully sealing the coverslip leads to condensation and pressure buildup causing bulging that will move cells and inhibit long-term automatic imaging. Static images were taken at room temperature 22°C, and long-term timelapses were taken at 30°C.

Images were acquired with a DeltaVision Core (Applied Precision, Issaquah, WA, USA) microscope using a 60× N.A. 1.4 PlanApo objective lens and a 12-bit Photometrics CoolSNAP HQII CCD. The system x–y pixel size is 0.109 μm. softWoRx v4.1 (Applied Precision, Issaquah, WA, USA) software was used at acquisition. Three-dimensional stacks were deconvolved with manufacturer provided OTFs using a constrained iterative algorithm. Excitation illumination was from a solid-state illuminator, and a proper polychromic mirror and filter set was used according to the individual or combined fluorophores. Appropriate excitation intensities and exposure times are available in the following section.

### Image processing and analysis

All image processing and analysis were done using the imaging software and plugin package ImageJ–FIJI (Schindelin *et al*. 2012). All foci counting was quantified using a computational algorithm based on uniform threshold per fluorescence channel as described by the light microscopy core facility at Duke University (https://microscopy.duke.edu/guides/count-nuclear-foci-ImageJ).

Foci-based colocalization analysis was performed using the ImageJ plugin JACoP–Manders coefficient (Bolte and Cordelières 2006). Colocalization was quantified using an observer set standardized threshold per replicate and per treatment vs nontreatment. 3D nucleolar volume was calculated using the nucleolar marker GFP-Nhp2 in a WT and Δ*swi6* background. Forty Z-stack segments with a.1-μm distance using a 100 × objective were taken. Light source intensity was set at 32% with an exposure time of .08 sec per image. Image stacks were 3D projected which was made into a 3D mask for further analysis. The ImageJ plugin 3D Objects Counter was used to calculate the 3D internal volume of the GFP-Nhp2 marker for the nucleolus (Bolte and Cordelières 2006).

Long-term timelapse videos of GFP-Nhp2 and Gar2-mCherry were taken every 10 min for a total of 180 min. Ten z-stack images were taken at each time point per channel with a z distance of .35 μm. Light source intensity was set at 32%, for .05 sec for mCherry, and 10% for .45 sec for GFP. mCherry and GFP images were bleach corrected using the ImageJ plugin bleach correction–histogram matching (Miura 2020). GFP images in 1,6-hexanediol-treated cells were not bleach corrected due to the lack of bright-enough GFP-Nhp2 signal. Images were stabilized in ImageJ–Fiji (Schindelin, J., *et al*. 2012) using the package “StackReg” by Philippe Thevenaz from the Biomedical Imaging Group at the Swiss Federal Institute of Technology Lausanne (Thevenaz *et al*. 1998).

### qPCR assay

Long-term growth in 1,6-hexanediol rDNA copy number ratio change was calculated via 18 s rDNA sequence to the *act1* gene. Genomic DNA was first extracted after the particular number of days’ growth in 1,6-hexanediol and rich media (YES) using phenol chloroform extraction (Forsburg and Rhind 2006). DNA concentration was calculated via a NanoDrop 1000 spectrophotometer (Thermo Scientific). Aliquots of 20 ng/μL were made, and both samples were stored at −20°C until used. qPCR was done using iTaq Universal SYBR Green Supermix (Bio-Rad) and a CFX96 Connect Real-Time PCR System (Bio-Rad). Approximately 20-uL samples were run with a final concentration of 1 ng/μL. Standard curves with a R^2^ > 0.98 were used for relative quantification. Final values were calculated as 18 s/*act1* gene ratios. Primer sequences were developed using Primer-BLAST (National Institute for Biotechnology Information). Primer sequences used were *18s*FWD 5′-ATT GGA GGG CAA GTC TGG TG-3′, *18s*REV 5′-CAG TCG ACC AGG CTC AAA-3′, *act1*FWD 5′-TGC TAC GTC GCT TTG GAC TT-3′, and *act1*REV 5′-GGA AAA GAG CTT CAG GGG CA-3′.

### Recombination assays

rDNA recombination rates were calculated via loss of a singular *ura4 +* gene located within the rDNA repeats. This strain and assay was performed via the protocol developed by Thon and Verhein-Hansen (2000). Centromere stability was observed via the minichromosome loss assay developed by Nakamura *et al.* (2008) and modified in Li *et al.* (2013).

## Results

### 1,6-Hexanediol inhibits *S. pombe* growth

**Fig. 1.**
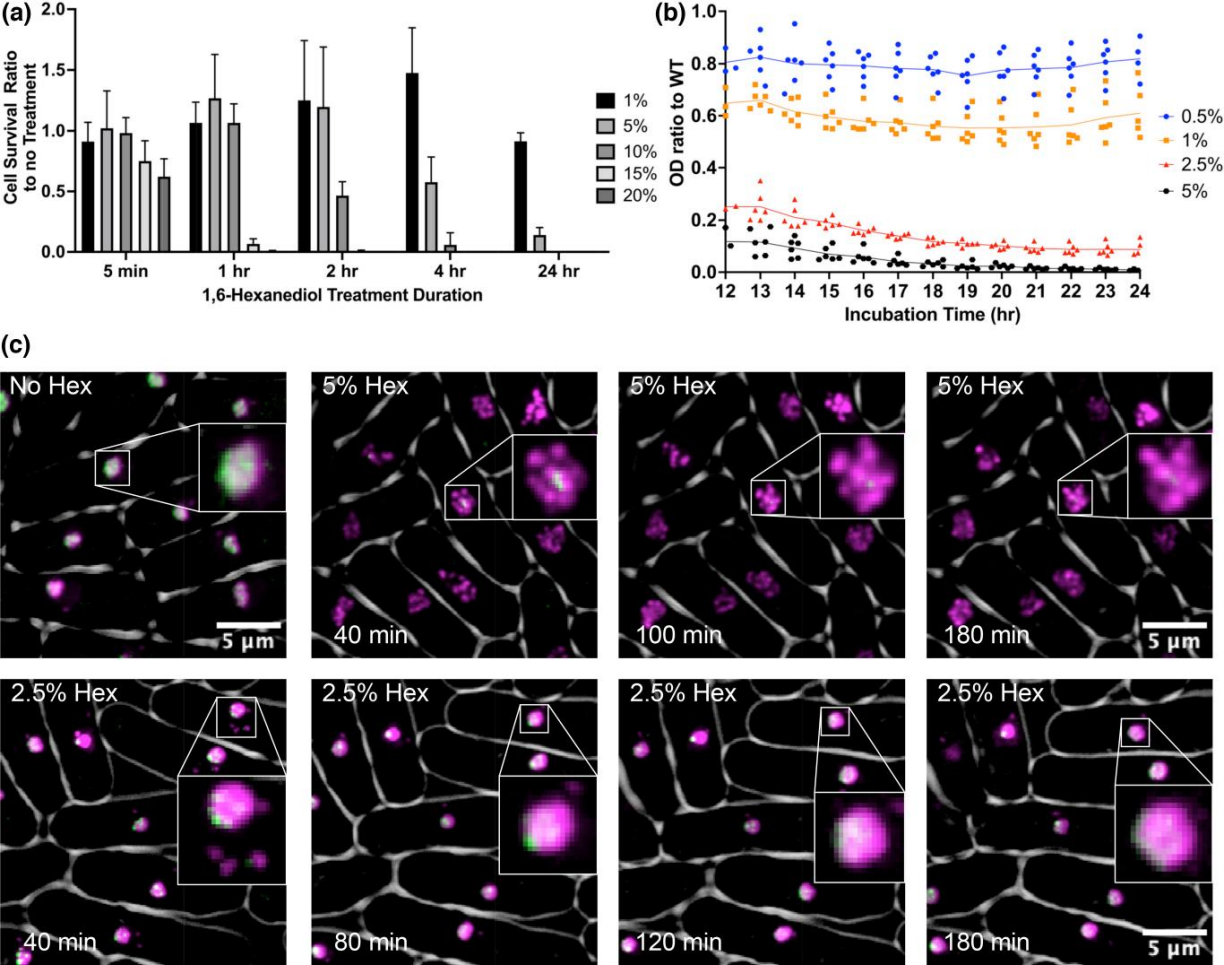
Characterization of 1,6-hexanediol affects on *S. pombe*. a) Cell survival ratio to no treatment. Cells were washed twice with PBS before plating. b) *S. pombe* growth rate ratio via OD 595 nm to WT in varying concentrations of 1,6-hexanediol. c) Representative images of 1,6-hexanediol treatment on nucleolar proteins Gar2-mCherry and GFP-Nhp2. Spindle pole body marker Sad1-mCherry was also added to visualize nucleolar localization within the nucleus. Visualization of Sad1-mCherry is usually only seen upon oversaturation due to Gar2-mcherry brightness. Gar2-mcherry and Sad1-mCherry are shown in false color as magenta and GFP-Nhp2 as green.

1,6-Hexanediol causes an acute loss of liquid–liquid phase separation (LLPS) via weak hydrophobic interaction (Ribbeck and Görlich *et al*. 2002) (Strom *et al*. 2017). We characterized its effects on fission yeast cell physiology, survival, and growth rate. Cells were incubated in rich yeast extract supplemented (YES) media with 1,6-hexanediol at 1, 5, 10, 15, and 20% (Fig. 1a) and plated at different time points for viability. After 5 min there was a slight decrease in cell survival at the highest concentrations of 15 and 20%, but we observed no effect at lower concentrations. However, after 1 h, there was a decrease in viability at concentrations greater than 10%; at 2 h, a similar decline was observed for concentrations above 5%. After 24 h, only the 1% 1,6-hexanediol-treated cells survived, with all other concentrations having a drastic decrease in cell survival. Thus, there is a dose and time-dependent decrease in *S. pombe* cell survival upon treatment with 1,6-hexanediol.

Complementing our viability analysis, we measured OD 595 nm to assess the growth rate of *S. pombe* cells in rich media. We took the ratio of OD value of the 1,6-hexanediol-treated cells vs our untreated WT strain (Fig. 1b and Supplementary Fig. 3). At 0.5% 1,6-hexanediol, the growth rate is decreased to around 80% of WT. Increasing concentrations correlate with further reductions in growth rates. These data confirm that treatment with 1,6-hexanediol causes a dose-dependent decrease in *S. pombe* growth rate.

### Effects on nucleolar domains

It has been established that the nucleolus is phase separated (Weber and Brangwynne 2015) (Feric *et al*. 2016). We examined whether 1,6-hexanediol affects cellular organization of the nucleolus in *S. pombe* using live cell microscopy. We looked at the nucleolus using two different tagged nucleolar proteins, nmt(41x):GFP-Nhp2 and Gar2-mCherry, and a spindle pole body (SPB) marker Sad1-mCherry. Nhp2 is part of a small nucleolar binding protein complex (Maiorano *et al*. 1999), while Gar2 is the ortholog of the human nucleolin protein (Gulli *et al.* 1995). In WT cells, Gar2-mCherry and GFP-Nhp2 overlap almost exactly with each other and show a diffuse nucleolar localization which is directly opposite the SPB (Fig. 1c).

We began by using an intermediate treatment concentration of 2.5% 1,6-hexanediol for 15 min. We then processed cells for imaging on pads also containing 2.5% 1,6-hexanediol. We observed a rapid change in Gar2-mCherry and GFP-Nhp2 localization as soon as imaging was started at 40 min. Gar2 separates from a single element into one large circular focus with a portion of cells having a few smaller satellite bubbles of variable sizes, while GFP-Nhp2 either forms much smaller puncta within the larger Gar2-mCherry regions or more generally diffuse localization. Over the time course, we could see a gradual decrease in the number of Gar2-mCherry foci into one large circular region usually containing either one bright GFP-Nhp2 focus or scattered smaller foci. At a higher dose of 5% 1,6-hexanediol, we observed Gar2-mCherry and GFP-Nhp2 disruption occurred as quickly as the lower concentration, but Gar2-mCherry showed far more scattered smaller circular bubbles (5–7), and little to no GFP-Nhp2 was observed. Over the timelapse the scattered bubble appearance of Gar2-mCherry was maintained; however there was a reduction in overall number of bubbles which coalesced into fewer larger bubbles (1–4) that maintained the near absence of GFP-Nhp2 (Fig. 1c).

Next, we tested to see how the 1,6-hexanediol-treated cells were able to recover normal nucleolar phase separation via WT Gar2-mCherry and GFP-Nhp2 localization. Cells were treated with either 2.5% or 5% 1,6-hexanediol, grown for 120 min, washed twice with 1 × PBS, resuspended in media for 5 min, and then processed for imaging. In both the 5 and 2.5% treated cells, there is a gradual resumption of normal Gar2-mCherry localization from the bubble like spread out circular shape of the 1,6-hexandiol treated phenotype to its more WT single ovicular shape. GFP-Nhp2 is much slower to resume its diffuse WT nucleolar localization. In the 5% treated cells, there is very little redistribution across the nucleolus, and its large foci-type localization is mostly maintained even out to 100 min. In the 2.5% treated cells, GFP-Nhp2 more readily redistributes diffusely across the nucleolus similar to WT by the end of imaging at 60 min (Supplemental Figs. 1 and 2).

### Phase separation stabilizes heterochromatic regions

**Fig. 2.**
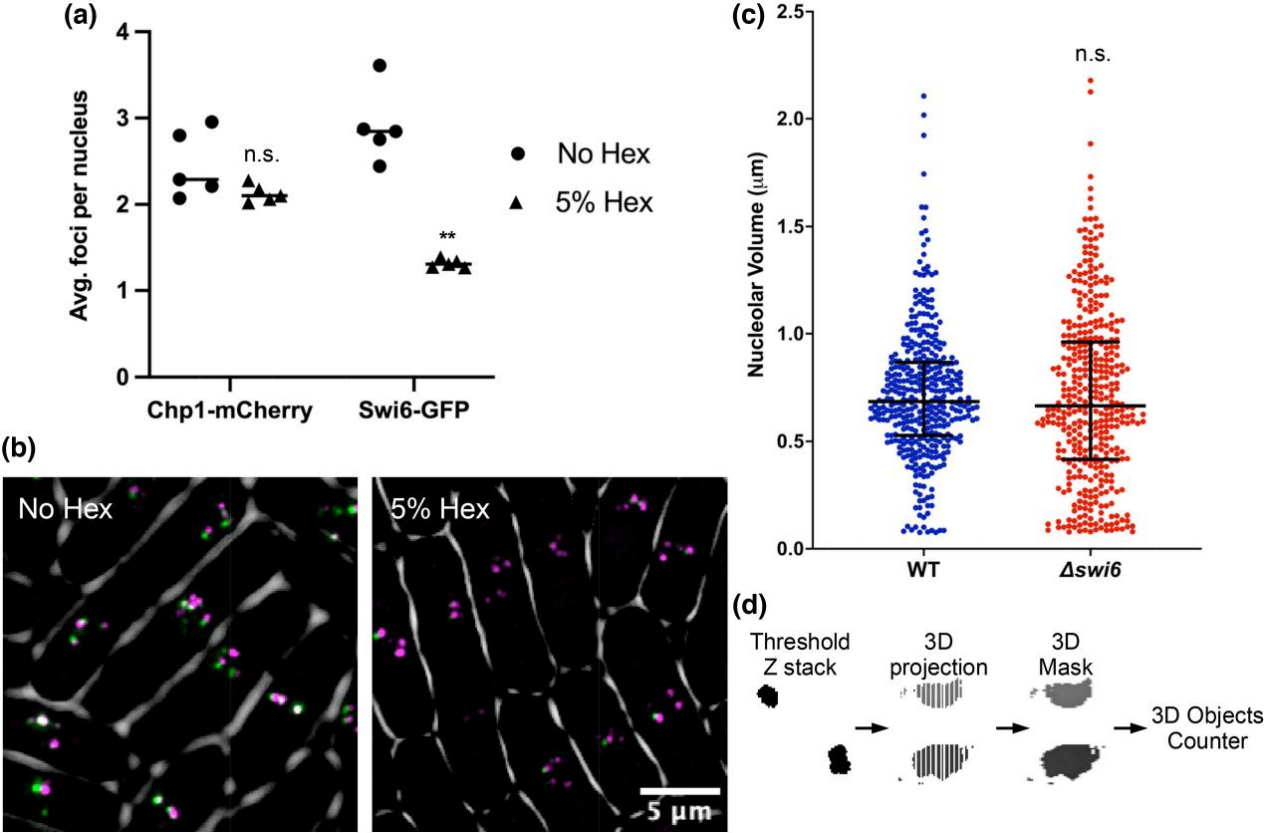
Phase separation stabilizes heterochromatic regions. a) Average number of Chp1-mCherry and Swi6-GFP foci per nucleus with and without 2-h 1,6-hexanediol treatment. (**P value < 0.01, Mann–Whitney U test) b) Representative images of 1,6-hexanediol-treated cells from a). False color was used, magenta represents Chp1-mCherry and green represents Swi6-GFP. Colocalization is white. c) 3D nucleolar volume of WT and Δswi6 using the nucleolar marker GFP-Nhp2. N = 3. (*P* value = 0.4771, Mann–Whitney U test) d) Workflow of 3D nucleolar volume image analysis.

Another region of the genome linked to phase separations is heterochromatin defined by the eukaryotic H3K9me3 binding protein HP1 (Larson *et al*. 2017; Matsuda *et al*. 2017; Strom *et al*. 2017; Tatarakis *et al*. 2017). Using live cell imaging, we looked at two of the major H3K9me3 heterochromatin binding proteins in fission yeast: the HP1 homologue Swi6-GFP which as discussed previously has been shown to phase separate in vitro and Chp1-mCherry part of the RITS complex responsible for heterochromatin binding/establishment, which has not been shown to have any phase separation capacity. Untreated cells have ± 2 foci of Chp1-mCherry and ±3 Swi6-GFP (Fig. 2b), which have been shown previously to correspond to the centromeres, telomeres, and mating-type regions associated with H3K9me heterochromatin (Ekwall *et al*. 1995; Cheutin *et al*. 2004; Petrie *et al*. 2005; Schalch *et al*. 2009). Upon treatment with 5% 1,6-hexanediol for 2 h, we observed a decrease in the number of Swi6-GFP foci to around 1 but no change in Chp1-mCherry. Thus, Swi6 foci localization is partially disrupted upon loss of phase separation.

Within the nucleolus roughly 50% of WT rDNA repeats are heterochromatinized at any point (French *et al*. 2003; Lindström *et al*. 2018). Thus, since we had seen such a drastic decrease in Swi6-GFP foci upon loss of phase separation via 1,6-hexanediol treatment, we hypothesized that loss of this protein could disrupt proper nucleolar structure. Using a Δ*swi6* background, we analyzed the 3D nucleolar volume using the nucleolar GFP-Nhp2 tag previously used. In WT cells the nucleolus maintained an average nucleolar volume of around. 5–1 μm, while cells lacking the Swi6 protein had a slight increase in nucleolar volume size distribution but no change in the average. These data indicate that while phase separation maintains proper Swi6 heterochromatin foci formation, its absence does not seriously disrupt the 3D structure of the nucleolus.

### 1,6-Hexanediol causes an increase in general genome instability

**Fig. 3.**
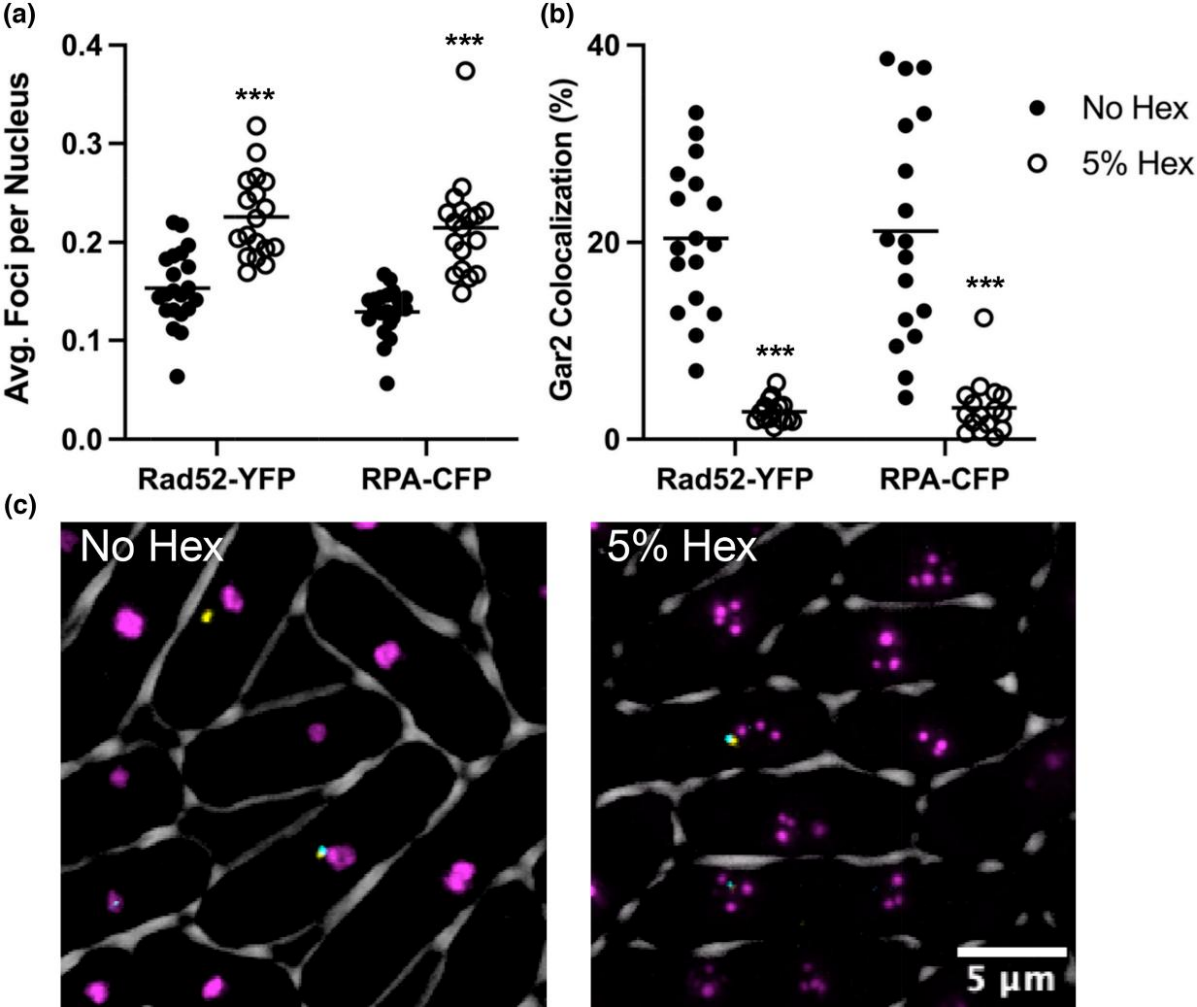
DNA damage protein foci increase but do not colocalize with Gar2 bubbles in 1,6-hexanediol. a) Average Rad52-YFP or RPA-CFP foci per nucleus with and without 1,6-hexanediol treatment. (***P < 0.001, Mann–Whitney U test) b) Manders correlation coefficient was used to calculate the 3D colocalization of Rad52 and RPA foci overlapping with Gar2 signal. (***P < 0.001, Mann–Whitney U test) c) 2D example images of Rad52-YFP, RPA-CFP, and Gar2-mCherry. Rad52-YFP is represented in false color as yellow, RPA-CFP in cyan, and Gar2-mCherry in magenta.

Since 1,6-hexanediol causes a drastic decrease in cell growth rate and survival and given its known roles in phase separation of heterochromatin regions and the nucleolus, we next examined whether loss of phase separation via 1,6-hexanediol caused any increase in general genome instability. In order to do this, we examined localization of the homologous recombination (HR) protein Rad52-YFP (Rad22) and the ssDNA binding protein RPA-CFP (Rad11). In untreated cells there was on average ∼0.15 foci of both Rad52-YFP and RPA-CFP per nucleus that often colocalized with the Gar2-mCherry nucleolar marker (Fig. 3a and b). After treatment with 5% 1,6-hexanediol for 4 h, both Rad52 and RPA foci increased to on average ∼0.2 foci per nucleus (Fig. 3a). Even though there was an increase in foci per nuclei, these foci did not colocalize with the disrupted Gar2-mCherry bubbles (Fig. 3b and c). These data suggest that 1,6-hexanediol-mediated loss of phase separation causes an increase in general genome instability seen by an increase in nuclear DNA damage protein foci.

**Fig. 4.**
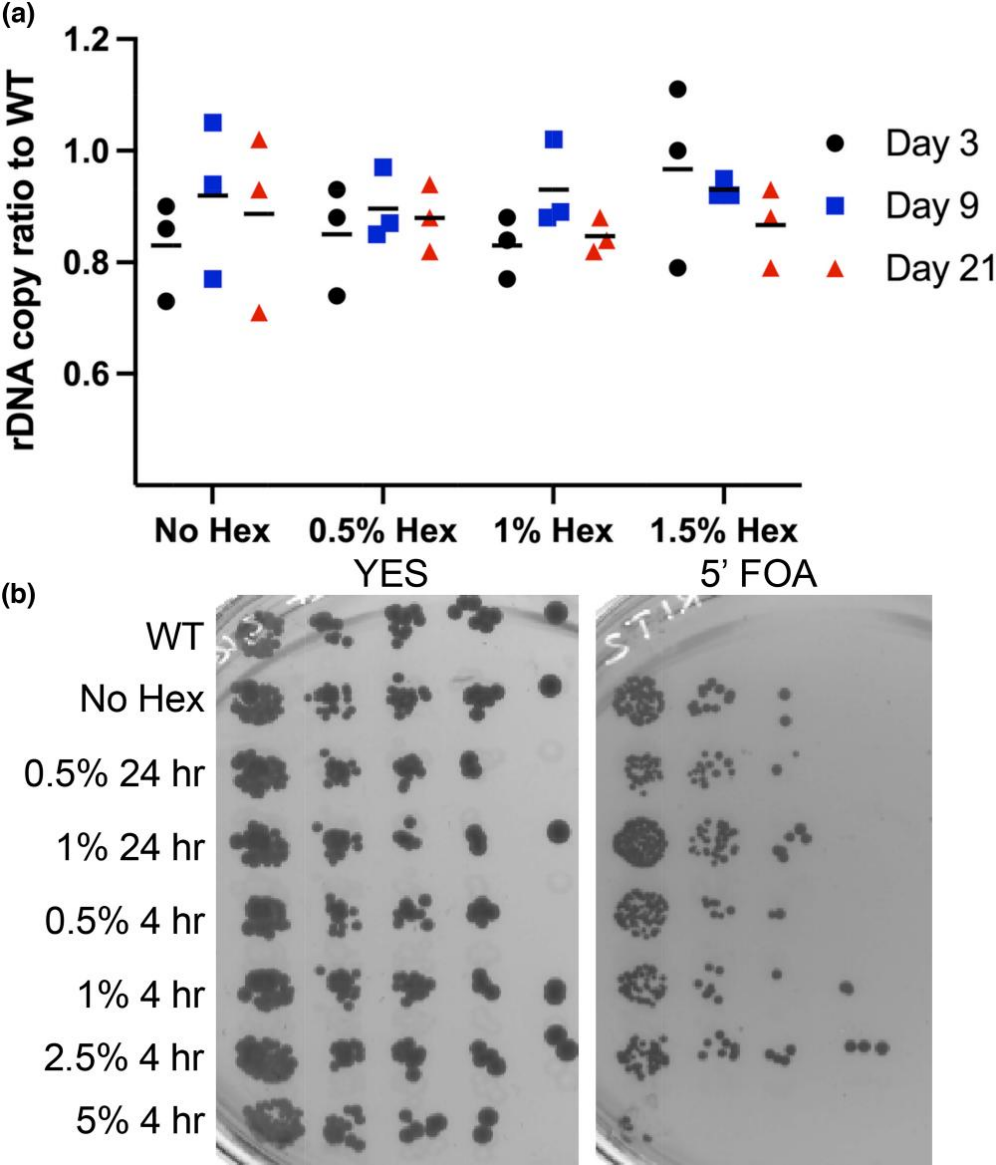
rDNA copy number and stability are not phase separation dependent. a) rDNA quantity ratio to WT using qPCR to the 18 s/*act1*. b) +ura4 gene loss from the rDNA array. Cells were grown in YES +/− 1,6-hexanediol and then plated on 5-fluoroorotic acid. Cells that are capable of growing have lost the + ura4 gene from the rDNA array. WT contains the native + ura4, while the + ura4 in the rDNA has no Hex.

### Stability of the rDNA repeats and centromere upon treatment with 1,6-hexanediol

Since 1,6-hexanediol causes an increase in general genome instability, we sought to identify what regions of the genome could be most affected. Since we saw a disruption of Gar2 and Nhp2 localization in 1,6-hexanediol, we investigated the copy number of the rDNA repeats. We used relative qPCR of 18 s rDNA copies per *act1* gene copy ratio to calculate the average rDNA ratio to WT per cell. For our long-term treatment group, cells were grown over a 21-day period. Samples were taken at days 3, 9, and 21 treated with 0.5, 1, and 1.5% or a no 1,6-hexanediol control. There was no change in average ratio rDNA copies to WT in any of the treatment groups (Fig. 4a). These results show that long-term low concentration 1,6-hexanediol treatment does not cause a substantial change in rDNA copy number repeats.

Even though the relative rDNA copy number via qPCR is maintained, we wanted to confirm if the instability of the repeats was increased yet the number of repeats remained stable. We next examined whether there was any difference in the loss rate of *ura4^+^* inserted into the rDNA array internal noncoding IGS sequence (Thon and Verhein-Hansen 2000). Upon plating on 5′FOA, any cell that has lost or silenced *ura4^+^* gene from the rDNA will survive on this drug. These cells were treated with either no 1,6-hexanediol, 0.5% or 1% for 24 h, or 0.5, 1, 2.5, or 5% for 4 h. A strain with no *ura4* + gene inserted in the rDNA was added as a control. Our results show that across all the treated and untreated groups there is no change in *ura4 +* loss compared to WT except for the cells treated for 4 h at 5% 1,6-hexanediol (Fig 4b). We are unsure of the cause of this decreased survival on 5′FOA. Three possibilities are that high-dose 5% 1,6-hexanediol was too toxic and thus the cells could not take further insult by the 5′FOA which is also toxic in itself, the 1,6-hexanediol caused a decrease in heterochromatin at the IGS of the rDNA allowing much higher rates of *ura4^+^* transcription, or loss of phase separation via 1,6-hexanediol caused partial permeabilization of the cell wall allowing a higher dose of 5′FOA to enter the cells compared to the other treatment groups. Overall, despite complications in the higher concentration group, these results confirm that 1,6-hexanediol does not cause an increase in rDNA instability compared with WT.

**Fig. 5.**
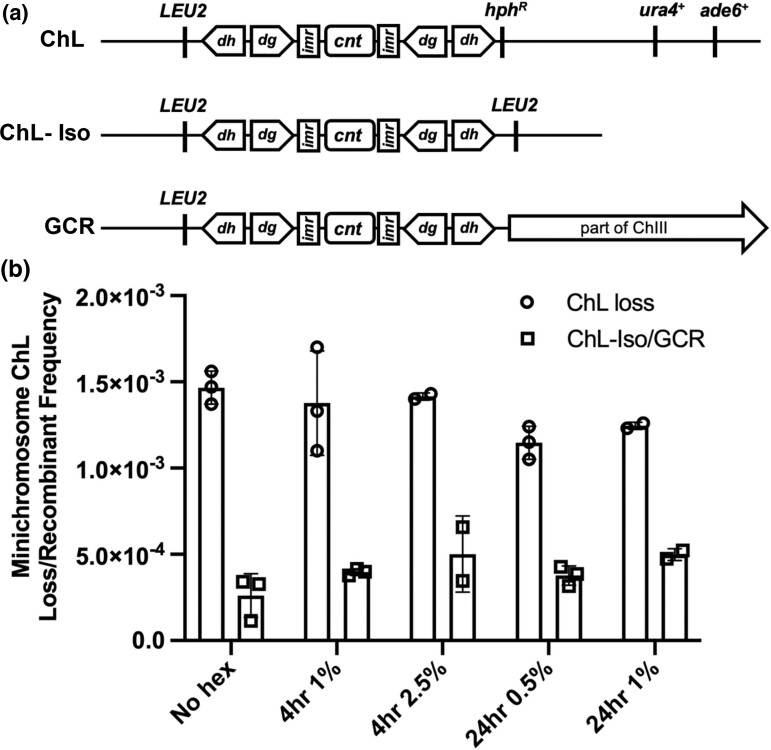
Centromere stability is independent of phase separation. a) Diagram of ChL from Nakamura *et al*. 2008, adapted from Li *et al*. 2013, and possible outcomes of minichromosome loss or ChL-Iso/GCR derivative formation. b) Minichromosome loss/recombinant frequency in 1,6-hexanediol.

We next examined the heterochromatic pericentromere, which is a major site for Swi6/HP-1 binding. We began by using a strain with a minichromosome originally derived from *S. pombe* chromosome 3. This strain contains multiple genetic markers that allow rapid identification of chromosome loss or gross chromosomal rearrangement (GCR) (Nakamura *et al*. 2008; Li *et al*. 2013) (Fig. 5a). Cells were treated with either 0.5% or 1% 1,6-hexanediol for 24 h or 1% or 2.5% for 4 h. We monitored genetic markers to observe if there were any changes in the stability of the minichromosome, including chromosome loss or gross chromosome rearrangement. Our results indicate that in all treated groups observed there is no increase in either GCR or minichromosome loss (Fig 5b). These data suggest that the increase in genome instability seen by an increase in DNA damage protein foci is not due to 1,6-hexanediol-mediated loss of phase separation destabilizing the centromere/pericentromere.

## Discussion

Phase separation has recently emerged as a major principle in the organization of the nucleus, allowing separation of chromatin domains and concentration of proteins without membrane-delimited organelles (Weber and Brangwynne 2015; Feric *et al*. 2016). Some forms of phase separation rely on weak hydrophobic binding and can be disrupted by treatment with the aliphatic alcohol 1,6-hexanediol (Romero *et al*. 2000; Ribbeck and Görlich 2002; Kroschwald *et al*. 2015; Uversky 2017). In this study, we investigated the consequences to fission yeast cells following treatment with 1,6-hexanediol. We observed a dose-dependent decrease in cell survival and growth rate even at low concentrations. At these low concentrations, we saw an increase in general DNA damage as measured by an increase in foci of DNA damage response proteins RPA and Rad52, consistent with a disruption in genome stability.

We assessed the effects of 1,6-hexanediol on regions of the genome that are known or presumed to be phase separated: the rDNA array and heterochromatin. We observed that the nucleolar markers Gar2 and Nhp2 were disrupted in treated cells, suggesting that proper nucleolar structure is disrupted. We also observed partial delocalization of the heterochromatin protein HP1-Swi6 from some of the foci where it is usually found but no change in another nonphase separating heterochromatin binding protein Chp1. These observations suggested that normal function of these repetitive HP1-specific bound heterochromatin domains might be impaired.

We assessed genome stability in the rDNA by examining the number of copies of rDNA repeats and observed no difference following 1,6-hexanediol treatment. Similarly, using a minichromosome with multiple markers, we determined the rates of chromosome loss or chromosome rearrangement, both of which are associated with loss of *swi6* (Li *et al*. 2013) and observed no changes. Thus, even though there may be disruption of phase separation in the nucleolus and pericentromere, we do not see consequences on genome stability. We hypothesize that an increase in genome damage seen via Rad52-YFP and RPA-CFP may be widespread and nonspecific due to many nuclear processes, many still yet discovered, relying on phase separation and via our inability to localize instability to two known phase-separated domains. Future studies could analyze relative foci location based on distance to the centromere via Sad1-mCherry and a nuclear periphery marker such as Ccr1N-GFP or more site-specific damage localization with techniques such as ChIP-seq to evaluate the sites of Rad52 and RPA binding.

Many studies have focused on using the phase-disrupting molecule 1,6-hexanediol to test the phase separation capability of various proteins (Ribbeck and Görlich 2002; Patel *et al*. 2007; Kroschwald *et al*. 2015; Peskett *et al*. 2018; Itoh *et al*. 2021; Ulianov *et al*. 2021). Our study suggests that its effects on live cells are broadly cytotoxic, and the genome instability and DNA damage that result from low levels of 1,6-hexanediol cannot be obviously linked to known phase-separated domains. More precise methods of targeting phase separation will be required to dissect its role in different domains.

## Supplementary Material

jkad123_Supplementary_Data

## Data Availability

The authors affirm that all data necessary for confirming the conclusions of the article are present within the article, figures, and tables. All raw data is available upon request from the corresponding author. Supplemental material available at G3 online.
