# Supplementary material for: Impact of 1,6-hexanediol on Schizosaccharomyces pombe genome stability
Source: G3 (Bethesda). 2023 Jun 7;13(8):jkad123. doi: 10.1093/g3journal/jkad123 (PMC10411564; doi:10.1093/g3journal/jkad123)
Supplement: jkad123_Supplementary_Data [file jkad123_supplementary_data.pdf]

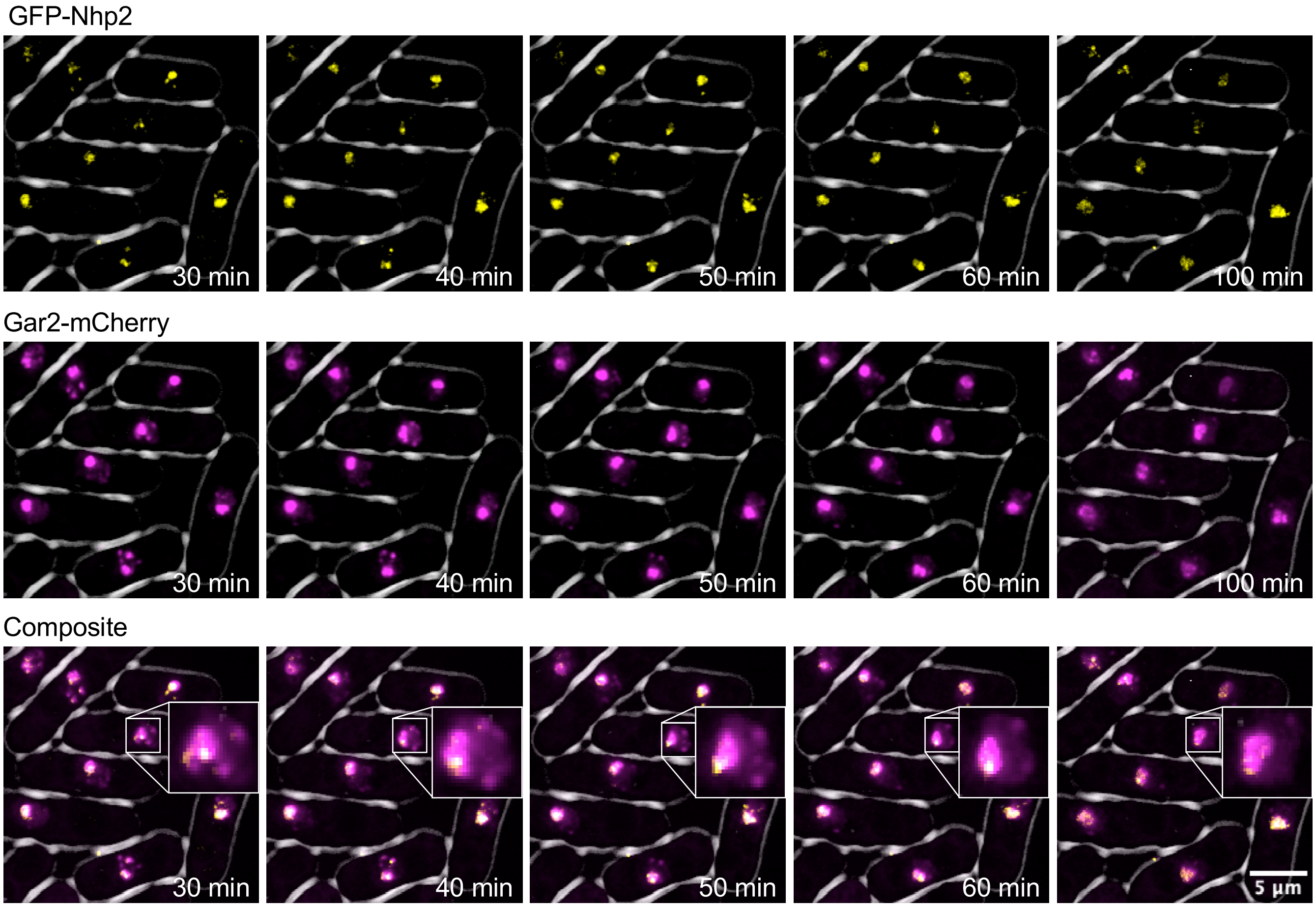

Supplemental Figure 1. Acute recovery from 5% 1,6-hexanediol
Cells were grown for 2 hours in 5% 1,6-hexanediol, washed two times with 1x PBS, and plated directly on nutrient pads. Images were taken every 10 minutes. GFP-Nhp2 is shown in false color as yellow and Gar2-mCherry and Sad1-mCherry are shown in magenta for ease of viewing.

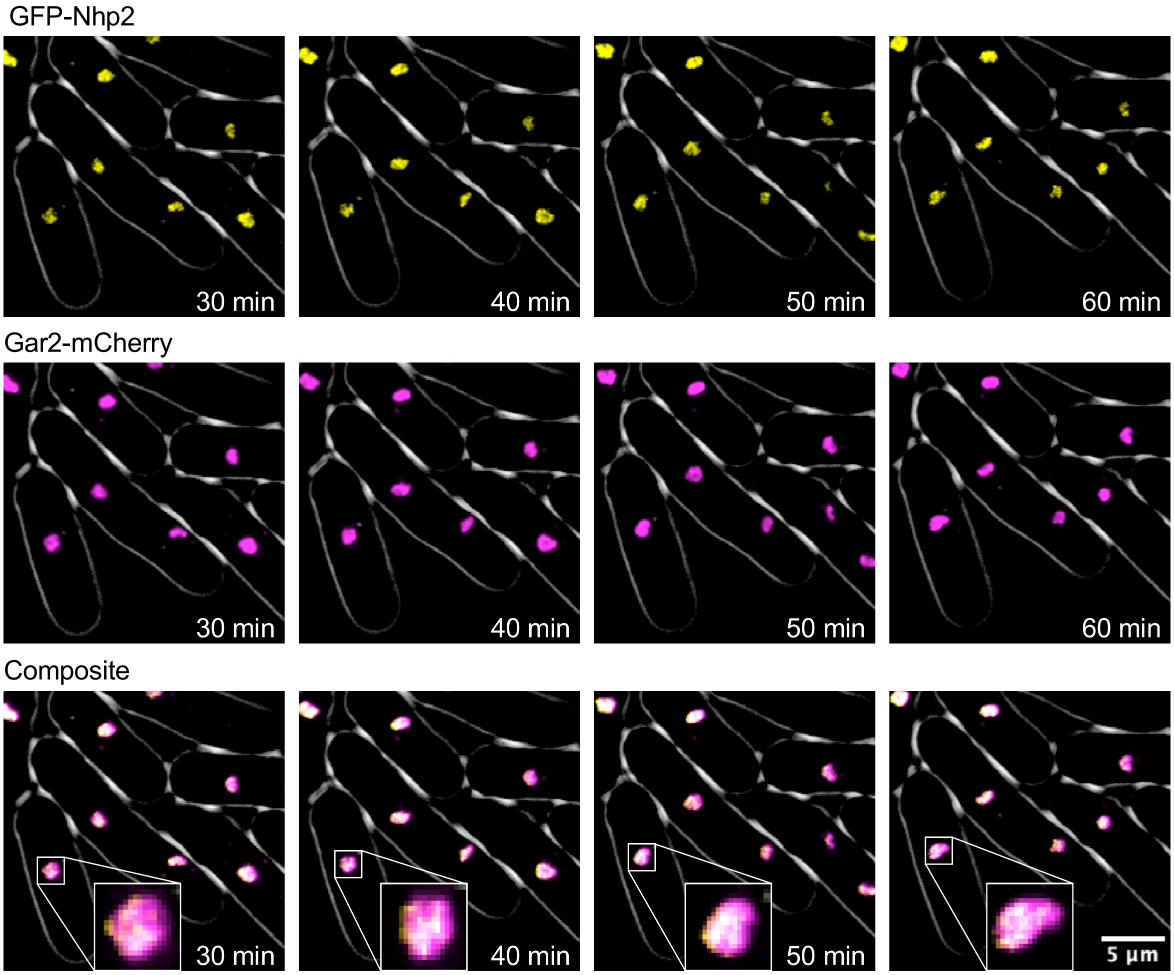

Supplemental Figure 2. Acute recovery from 2.5% 1,6-hexanediol
Cells were grown for 2 hours in 2.5% 1,6-hexanediol, washed two times with 1x PBS, and plated directly on nutrient pads. Images were taken every 10 minutes. GFP-Nhp2 is shown in false color as yellow and Gar2-mCherry and Sad1-mCherry are shown in magenta for ease of viewing.

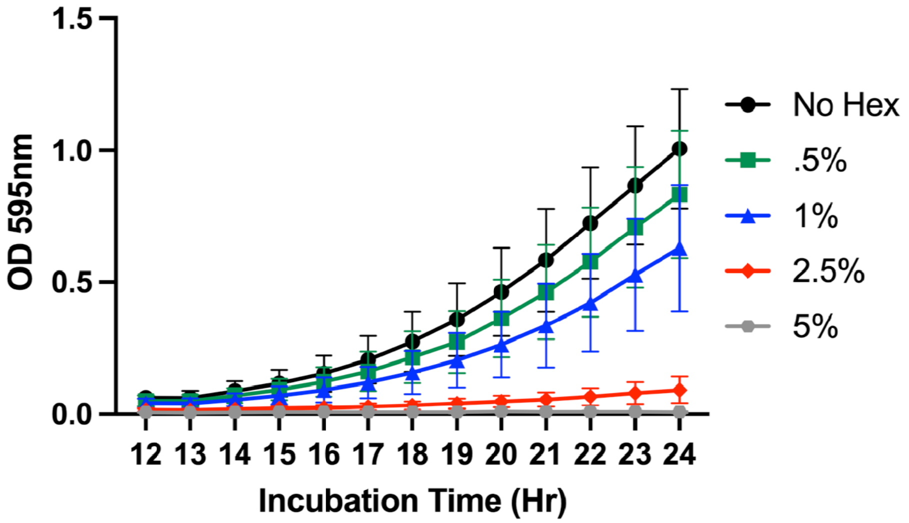

Supplemental Figure 3. *S. pombe* raw growth rate in 1,6-Hexanediol
Growth rate of *S. pombe* cells in varying concentrations of 1,6-hexanediol. OD was measured at 595nm and raw values were plotted and error bars represent SD, N=6.
